# 3D cell culture based on artificial cells and hydrogel under microgravity for bottom-up microtissue constructs

**DOI:** 10.3389/fbioe.2022.1056652

**Published:** 2022-11-14

**Authors:** Ruimin Long, Linrong Shi, Peng He, Jumei Tian, Shibin Wang, Jun Zheng

**Affiliations:** ^1^ College of Chemical Engineering, Huaqiao University, Xiamen, China; ^2^ Engineering Research Center of Artificial Organs and Materials, Ministry of Education, Jinan University, Guangzhou, China; ^3^ Institute of Pharmaceutical Engineering, Huaqiao University, Xiamen, China; ^4^ Engineering Research Center of Fujian University for Stomatological Biomaterials, Xiamen Medical College, Xiamen, China; ^5^ Fujian Provincial Key Laboratory of Biochemical Technology, Huaqiao University, Xiamen, China; ^6^ College of Materials Science and Engineering, Xiamen University of Technology, Xiamen, China

**Keywords:** tissue engineering, bottom-up, artificial cell, microgravity culture, hydrogel

## Abstract

The use of hydrogel as a filling medium to recombine dispersed microencapsulated cells to form an embedded gel-cell microcapsule complex is a new idea based on bottom-up tissue construction, which is benefit for cell distribution and of great significance for tissue construction research *in vitro*. In this experiment, sodium alginate and chitosan were used as the main materials, rat normal liver cell BRL-3A was used as the model cell to prepare “artificial cells”. Silkworm pupa was used as raw material to extract silk fibroin solution, which was prepared by ultrasound to be the silk fibroin gel; silk fibroin hydrogel-microencapsulated hepatocyte embedded complex was then prepared by using silk fibroin gel as filling medium; the complex was cultured under three modes (static, shaking, and 3D microgravity), and the tissue forming ability of rat hepatocytes was investigated. The results showed that the microgravity culture condition can enhance the cell proliferation and promote the formation of cell colonies in the microcapsules; silk fibroin can form an embedded gel-cell microcapsule complex with microencapsulated cells, which provided mechanical support for the structure of the composite. We hope that this bottom-up construction system will have potential applications in the fields of cell culture and tissue construction.

## Introduction

Compared with two-dimensional culture, three-dimensional culture enables cells to simulate tissue-like structures more effectively and provide optimal simulation microenvironment for cell growth. Besides, microgravity stimulates cell-cell and cell matrix interactions, which promotes the formation of three-dimensional clusters and promotes cell proliferation. For example, Costantini D et al. (Costantini et al., 2019) studied the effects of microgravity on human bile duct trunk/progenitor cells (HBTSCs) and found that stem gene expression in HBTSCs cultured in SMG microgravity conditions was significantly increased compared with HBTSCs cultured under normal gravity control conditions. Zhou X et al. (Zhou et al., 2019) fabricated microencapsulated cells through microcapsules loaded with C5.18 chondrocytes alginate/chitosan prepared by a high-voltage electrostatic method. It could be found from the cell viability (cell proliferation) cell proliferation (AO/EB staining) and cell viability (CCK-8 assay) results that microencapsulated cells had a better cell proliferation under 3D micro-gravity microgravity conditions using bFGF than under 2D conditions (including static and shaking conditions). Mattei C (Mattei et al., 2018) studied the rotating cell culture system and found that microgravity basically supports the generation and maintenance of neurological organs derived from human embryonic stem cells, and can be used as a model for human brain development. In addition, He L et al. (He et al., 2016) confirmed that microgravity conditions promote the proliferation of human dental pulp stem cells compared with normal gravity conditions, thereby effectively forming functional tissues and promoting dental tissue engineering. Other publications also revealed that 3D microgravity culture is benefit for cell propagation (Farid et al., 2018; Sapudom et al., 2021; Zou et al., 2022).

Besides, microencapsulation technology is also used for cell culture. Microcapsules can immobilize plant, microbial or animal cells, as well as enzymes, coenzymes or proteins, in a polymeric hydrogel membrane (Li et al., 2020), which has selective permeability and immunoisolation ability. Only oxygen, nutrients and metabolites can be transported through the membrane, while the IgG is stopped outside. The microcapsules embedded with living cells are also called “artificial cells”, which usually contain biological substances such as cells, DNA, RNA, and protein encapsulated in semipermeable membranes or other compartments. At present, cell microencapsulation technology is considered to be a viable treatment for the therapy of neurological and endocrine diseases (Montanucci et al., 2013). It can also be used for oral probiotic (Mawad et al., 2018) or hyperlipidemic therapy (Song et al., 2017). Furthermore, Zhang X et al. (Zhang et al., 2017) investigated the role of active oyster peptide extract (AOPS) microcapsules formed by microemulsion gelling to prolong their biological activity. The results showed that the AOPS microcapsule can effectively reduce IL-1β and TNFα and increase the levels of ACP and lysozyme for inflammatory cytokines.

Today, tissue engineering has attracted considerable attention. Compared with the traditional top-down approach, which may have the problem of cell distribution to form an organizational structure with complex microstructure features (Luo et al., 2019), modular tissue engineering, also called bottom-up way, is to construct a pseudo-ecological micro-structural unit and then assemble to form a large-scale organization (Nie and Takeuchi, 2018). Okudaira T et al. (Okudaira et al., 2017) used a bottom-up approach to prepare 3D liver tissue from hepatocyte spheroids covering human umbilical vein endothelial cells and NIH/3T3 cells, which had higher liver function activity and higher cell density, and hepatic vascular endothelial cells formed a regular network structure in liver tissue. Luo C et al. (Luo et al., 2019) compared the performance of top-down and bottom-up construction strategies *in vivo*, and the comparative studies showed that bottom-up construction strategy was more effective in the treatment of bone defects *in vivo*.

**SCHEME 1:**
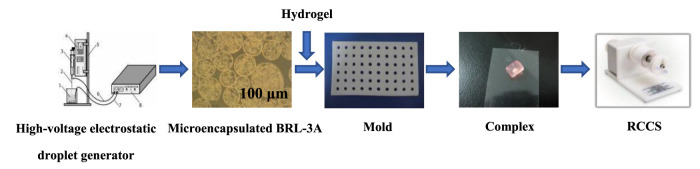
The complex was formed by the hydrogel and the microencapsulated BRL-3A cells prepared by the high-voltage electrostatic generator, and then was subjected to tissue construction under microgravity.

Therefore, to solve the problem of cell distribution in scaffold and promote the cell growth, a bottom-up strategy based on microcapsule and modular tissue engineering was proposed (Scheme 1). Microcapsules were prepared with sodium alginate and chitosan to encapsulate rat normal liver cell BRL-3A to form “artificial cells”; silk fibroin was used as the filling medium to fix the shape of the obtained silk fibroin hydrogel-artificial cell complex considering that silk fibroin is a commonly available natural biopolymer with suitable properties such as mechanical strength, elasticity, biocompatibility and controllable biodegradability, and has been widely used in tissue regenerations (Kundu et al., 2013; Gomes et al., 2022). Three different culture modes, including static, shaking, and 3D microgravity, were applied to primarily investigate the cell growth and functions.

## Materials and methods

### Materials

BRL-3A normal rat liver cells were purchased from Shanghai cell bank of Chinese Academy of Sciences, (Shanghai, China). High glucose DMEM medium and trypsin 1:250 were obtained from GIBICO Inc. (United States). Sodium alginate (ALG, A2158), acridine orange (AO) and ethidium bromide (EB) were provided by Sigma Inc. (United States). Chitosan (Chi, M.W. 1 × 10^5^ Dalton) was purchased from Golden Shell Biotechnology Co., Ltd. (Zhe Jiang, China). Albumin assay kit and urea assay kit were purchased from Zhongsheng Beikong Biological Co., Ltd. (Shanghai, China). HE kit was obtained from Plylai Biotechnology Co., Ltd. (Beijing, China). EDTA was purchased from Sinopharm Chemical Reagent Co., Ltd. (Shanghai, China).

### Preparation of microencapsulated cells

Preparation of microencapsulated cells using high-voltage electrostatic droplet generator (customized by Shanghai University of Technology) was conducted referring the method published elsewhere (Kundu et al., 2013) (Zhou et al., 2018). Briefly, adherently grown BRL-3A cells were digested with trypsin solution and mixed with 1.5% (w/v) sodium alginate solution (previously filtered through 0.8, 0.45, 0.22 μm filter). The volume ratio of the suspension to the Alg solution was 1:4. The cell suspension was adjusted to 1 × 10^4^/ml and dropped into 1.5% (w/v) calcium chloride solution through a high-voltage electrostatic droplet generator (parameter setting: 7.5 kV, 20 mm/h) to form Alg beads embedded with cells. The obtained beads were coated with a filter-sterilized chitosan solution for 8 min to obtain alginate/chitosan microcapsules, which were then suspended in 0.15% (w/v) Alg solution to form ACA microcapsules (Gomes et al., 2022) (Liu et al., 2015), and the core was liquefied with 1.6% (w/v) sodium citrate. The collected microcapsules were washed with saline, and placed in an incubator containing 5% CO2 at 37°C. The prepared microencapsulated cells were respectively cultured in three modes of static (24-well plate), shaking (Constant temperature incubator shaker, Weide Technology Co., Ltd., Xiamen, China) and microgravity culture system (RCCS-1 rotary microgravity incubator, Yituo Biological Instrument Co., Ltd., Shanghai, China).

### Preparation of silk fibroin hydrogel-microencapsulated cell complex

**SCHEME 2:**
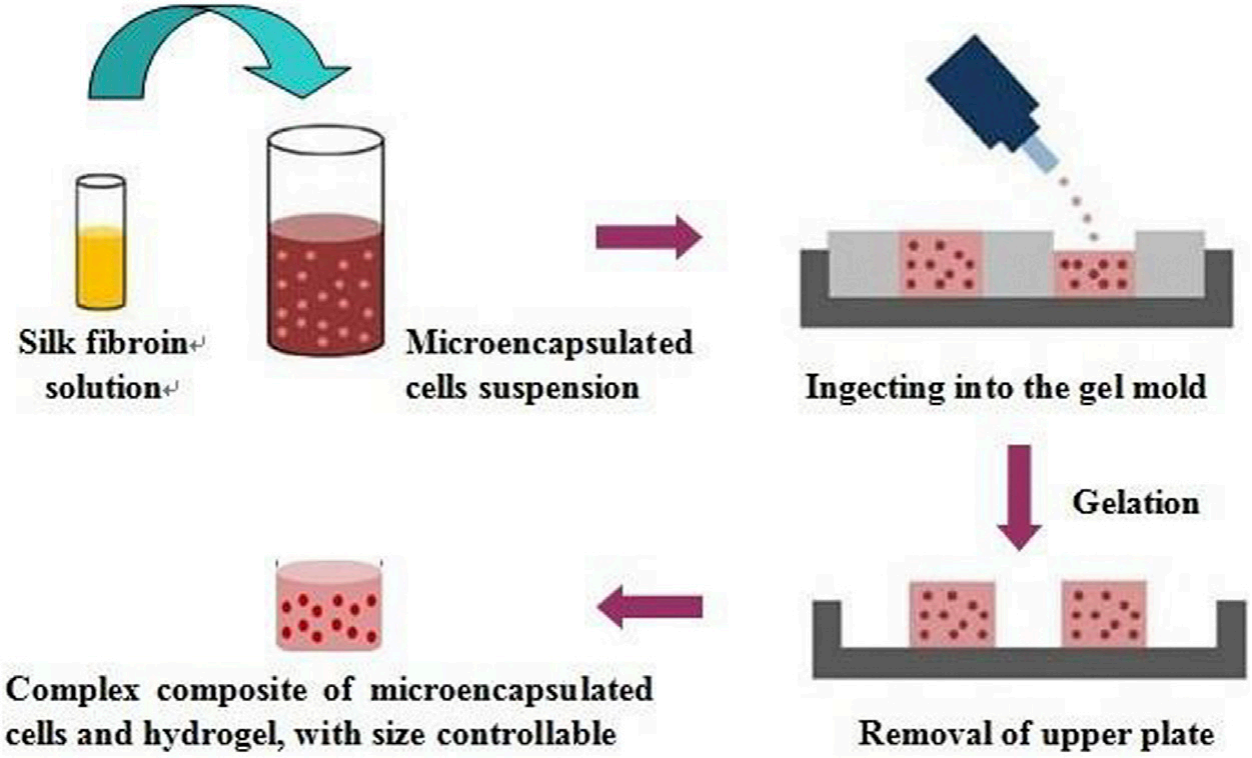
The preparation process of the silk fibroin gel and cell microcapsulation composites.

As shown in Scheme 2, firstly, silk fibroin aqueous solution was prepared. Briefly, silk fibroin solution was obtained by degumming, dissolving, dialysis, and concentration. Then the solution was diluted to 5% (w/v), and silk fibroin gelling solution was prepared at ultrasonic power of 200 W and time of 45s. The microcapsules and the solution were mixed at a volume ratio of 1:2. The resulting mixture was kept at room temperature for 5 min to obtain silk fibroin gel-microcapsule complex. The prepared silk fibroin-microencapsulated cell complex was respectively cultured in three modes of static, shaking and microgravity culture system.

### AO/EB staining

The suspension of 200 μL microcapsulated cells was mixed with 10 μL AO/EB mixture and incubated for 30 s. Take one drop of the above staining solution and drip it onto a clean glass slide. The sample was immediately observed under confocal laser microscopy (Zeiss, Germany) and 200 × objective microscopy (Zhou et al., 2018) (Zhao and Zhang, 2018).

### Determination of albumin secretion

The albumin content of BRL-3A cells in microencapsulated cells and complexes was measured at predetermined times according to the instructions of the albumin assay kit (Zhongsheng Beikong Biotechnology Co., Ltd., Shanghai, China) provided by the company.

### Determination of urea synthesis

According to the instructions of the urea detection kit provided by the company (Zhongbei Control Biological Co., Ltd., Shanghai, China), the urea content secreted by microencapsulated cells and BRL-3A cells in the complex was determined respectively at predetermined times.

### Statistical analysis

Results were expressed as mean ± standard deviation. Each measurement reported was based on duplicate analysis of at least three independent experiments.

## Results and discussion

### Microencapsulated BRL-3A cells and their activity

**FIGURE 1:**
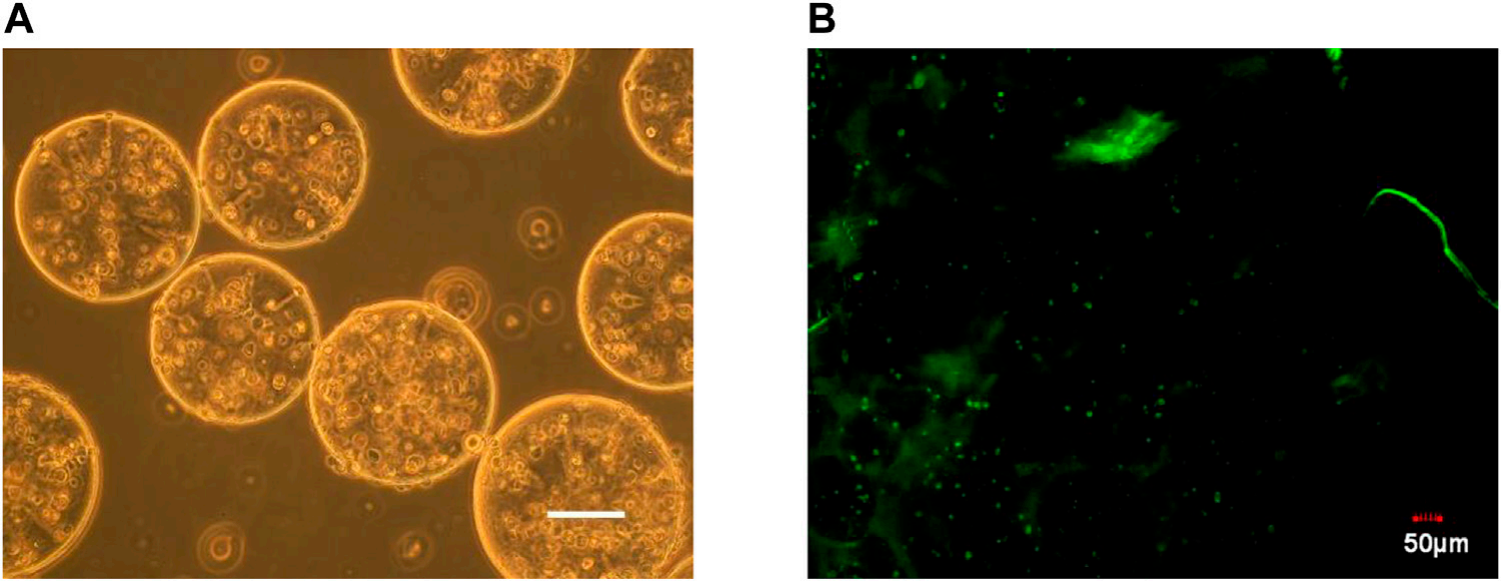
Microscopy photograph **(A)** and confocal laser scanning microscope image **(B)** of microencapsulated BRL-3A cells. (×200, left bar: 100 μm).

It can be seen from Figure 1 that the prepared calcium alginate/chitosan microcapsules were intact in morphology, good in sphericity, smooth in surface, non-adhesive between capsules, and well dispersed. The cells distributed well within the microcapsules. By AO/EB fluorescence staining, it can be clearly seen under the laser confocal microscope that the BRL-3A cells in the microcapsules were almost green, indicating that the cells in the capsule were basically living cells, and the survival rate was high, thereby confirming that the microcapsules did not affect cell survival.

### Culture of microencapsulated BRL-3A cells in three culture systems

**FIGURE 2:**
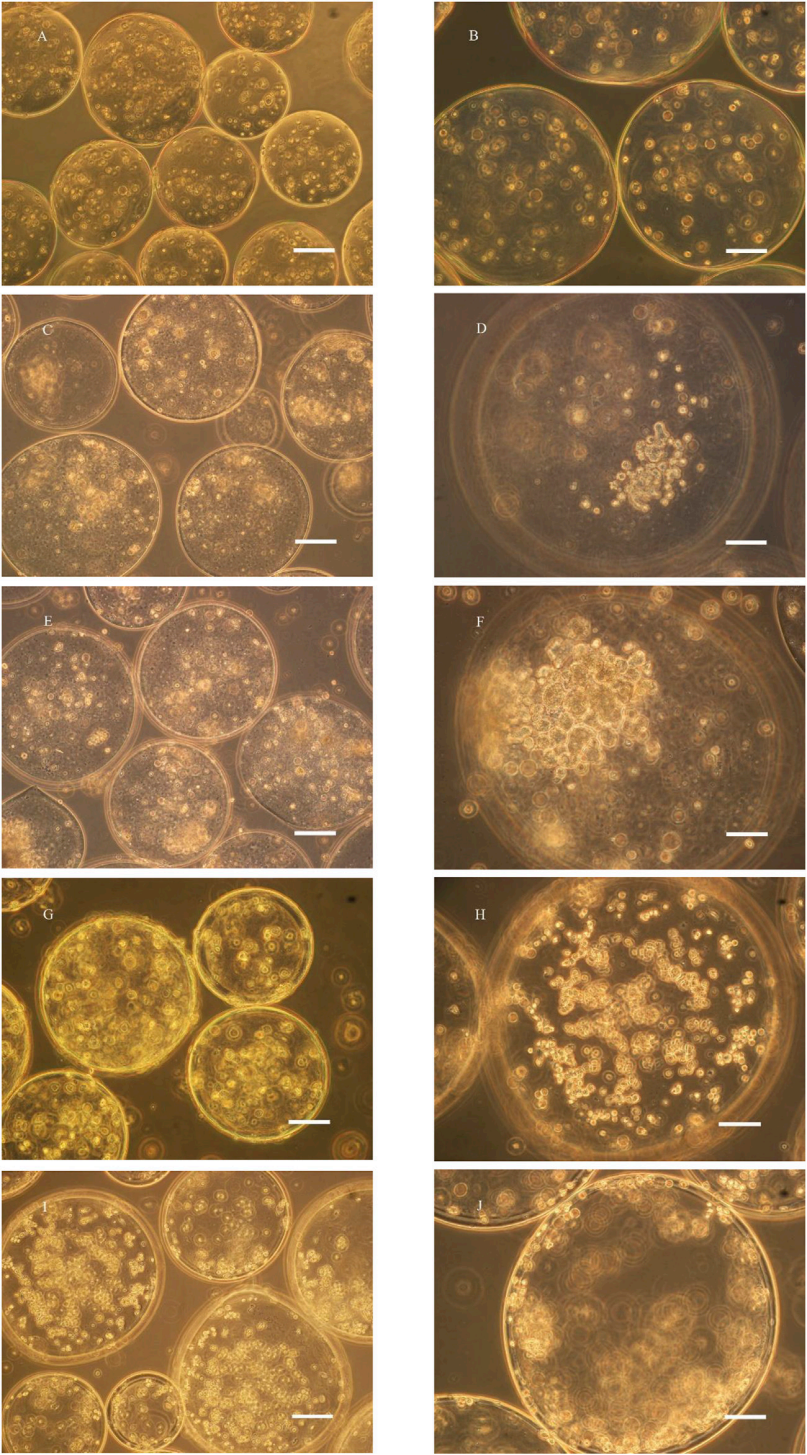
Microscopy photograph of microencapsulated BRL-3A cultured in rotary **(C–F)**, static **(G, H)** and shaking **(I, J)** cell culture system (left: ×200, bar: 100 μm; right: ×400, bar: 50 μm; **(A, B)**: 0 d; **(C, D)**: 7 d; **(E–J)**: 14 d).

The microencapsulated BRL-3A cells were cultured in a simulated microgravity culture system for up to 2 weeks. After one week of culture, the cells began to form a spherical shape with a diameter of about 50 μm, and the morphology and size were irregular, as shown in Figure 2D. After two weeks of cultivation, as shown in Figure 2E, the size of the sphere increased gradually, and a small cell mass of 30–50 μm in diameter formed a dense sphere having a diameter of 100–150 μm. Compared with the cell growth in the simulated microgravity culture system, there was no obvious cell colony formation after 2 weeks of static culture of the cells in the microcapsules, and only some cells aggregated toward the edge of the microcapsules (Figure 2H). Under shaking culture conditions, the cells in the microcapsules also did not form cell colonies in the central part, but there was obvious edge aggregation. Most of the cells aggregated toward the edge of the microcapsules, and a small number of small cell colonies were formed (Figure 2J).

In the study of Zhang (Liu et al., 2015) (Zhang et al., 2006), polylysine was used as a membrane material, and human breast cancer cells and chronic myeloid leukemia cells were embedded with sodium alginate, and cultured with an inducer in a static environment, and formed two weeks later. The cell colonies were about 180 and 70 μm in diameter, and the cell colonies were dense overall, and the cells therein had higher activity. In contrast, microencapsulated BRL-3A cells in static culture failed to express cell colonies after two weeks, while similar results were obtained under simulated microgravity culture. Compared with tumor cells, normal somatic cells have certain difficulties in the formation of tissue-like colonies, and better experimental results can be obtained in the culture system of this experiment, thus highlighting the effectiveness of simulated microgravity culture system in tissue construction.

### Culture of BRL-3A cells in 3D microgravity culture system

**FIGURE 3:**
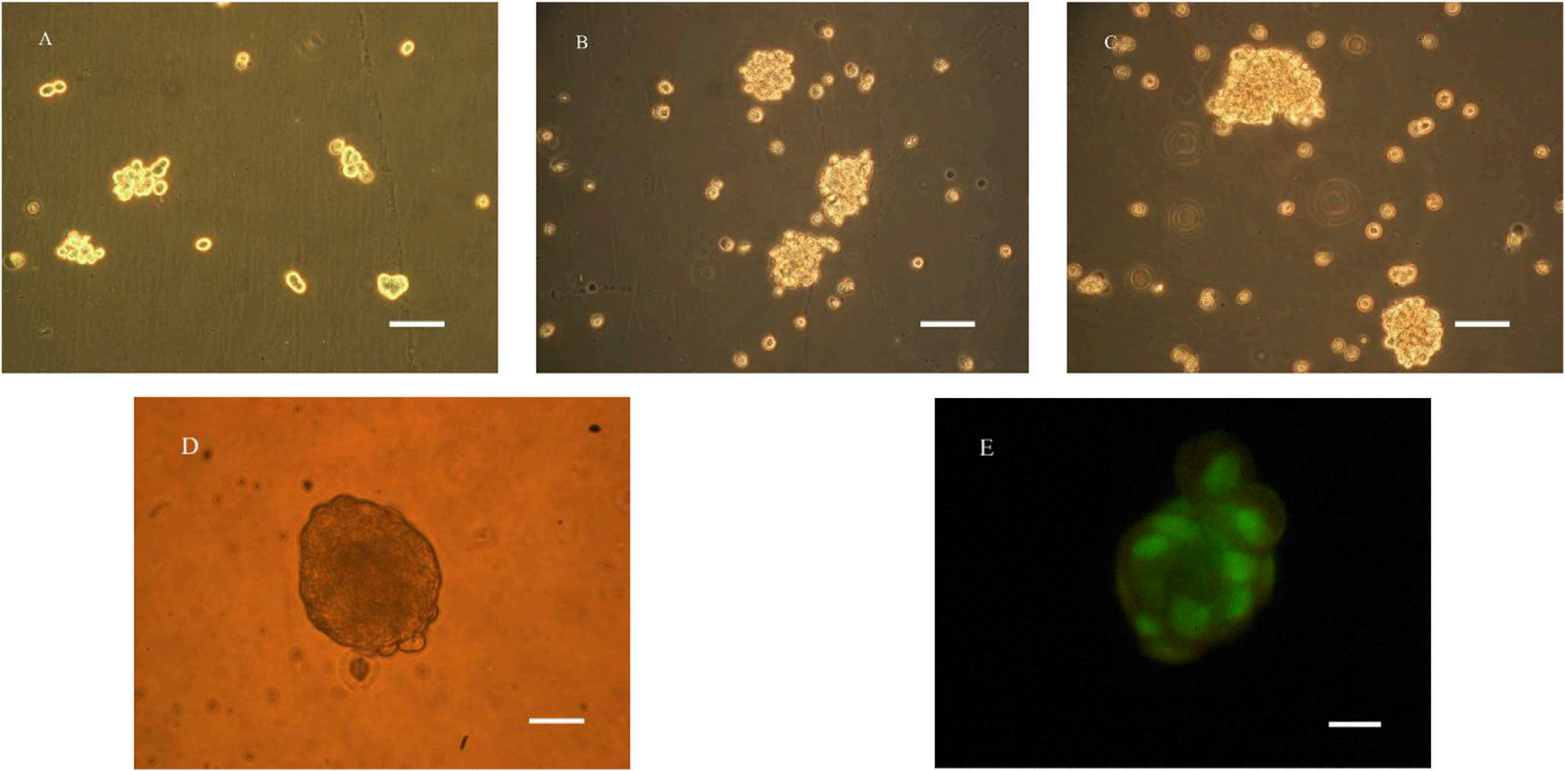
Microscopy photograph **(A–E)** and confocal laser scanning microscope image **(E)** of BRL-3A cultured in RCCS **(A)**: 1d, **(B)**: 4d, **(C–E)**: 7d; **(A–C)**: ×200, bar:100 μm; **(D, E)**: ×400, bar:50 μm).

To further verify the ability of cells to form aggregates under microgravity conditions, we cultured BRL-3A cells in a microgravity system alone as shown below:

Under RCCS culture conditions, liver cells did not attach to the wall and spread, but remained suspended and round. As can be seen from Figure 3A, after 24 h, the liver cells began to form a globular shape, and the shape and size were irregular. After one week of cultivation, as shown in Figure 3C, the size of the globule gradually increased, and a small cell mass of 30–50 μm in diameter formed a dense sphere with a diameter of 100–150 μm. It can be seen by fluorescence staining that the cell colony presented good cell survival phenomenon.

### Determination of albumin and urea in microencapsulated BRL-3A cells in three culture systems

**FIGURE 4:**
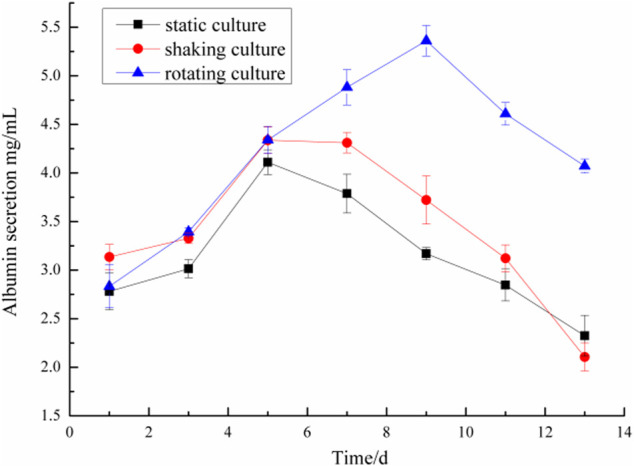
Comparison of albumin secretion of microencapsulated BRL-3A.

**FIGURE 5:**
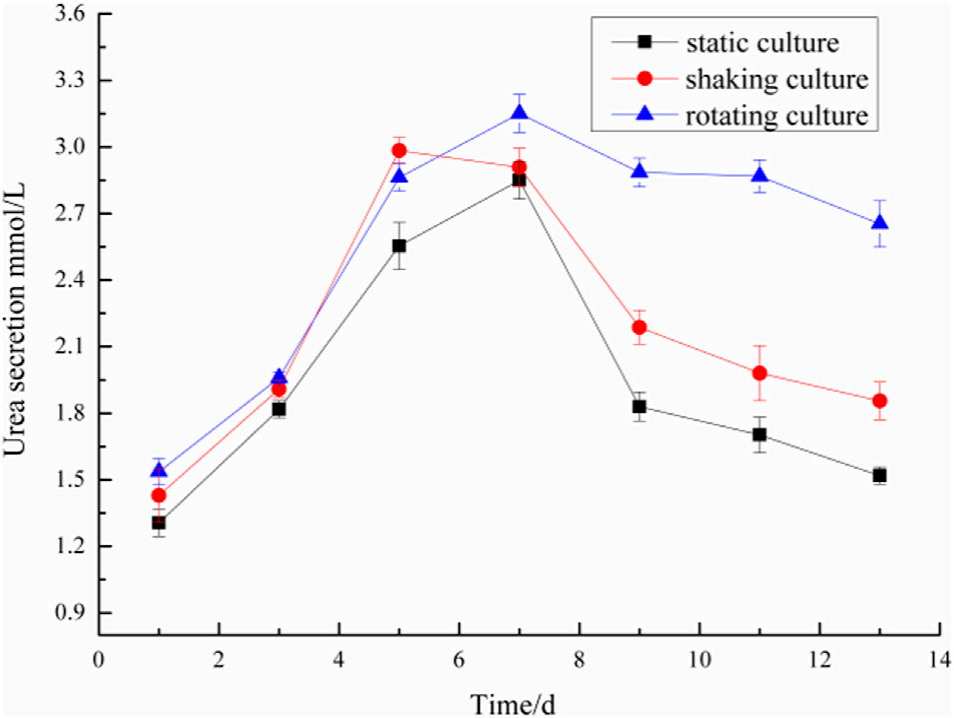
Comparison of urea secretion of microencapsulated BRL-3A.

Microencapsulated BRL-3A cells were cultured in three ways: static, shaking, and simulated microgravity. Clinically, albumin and urea are main indexes to examine the function of liver. Thus, the metabolism of hepatocytes was examined by detecting the albumin and urea content in the culture medium. The results were shown in Figures 4, 5.

On the first day, the albumin content in the culture medium was about 3.0 mg/ml and the urea content was about 1.4 mmol/L in the three culture modes. With the prolongation of the culture time, especially after 5 days, the metabolic properties of the microencapsulated hepatocytes under the simulated microgravity culture system were different. In the 5th day, the content of albumin synthesized by the three groups of microencapsulated hepatocytes increased, and the albumin content of microencapsulated hepatocytes in the simulated microgravity culture system increased significantly; in the 5th to 13th days, the amount of albumin secreted by microencapsulated hepatocytes in static and shaking culture systems was reduced compared with the previous days, but the microencapsulated hepatocytes under simulated microgravity culture system still showed strong albumin synthesis ability, and the albumin synthesis ability decreased until 10 days later.

In the 2nd to 7th days, the urea content of microencapsulated hepatocytes increased in the three medium culture mode, but after 7 days, the ability of microencapsulated hepatocytes to secrete urea in the static and shaking culture system decreased significantly and the amount of urea secretion decreased; in the 8th to 13th days, the ability of microencapsulated hepatocytes to secrete urea under simulated microgravity culture system decreased, but still maintained a high level. These results indicated that microencapsulated hepatocytes still had strong metabolic functions after long-term culture under simulated microgravity culture system.

### Preparation of silk fibroin-microencapsulated cell complex

**FIGURE 6:**
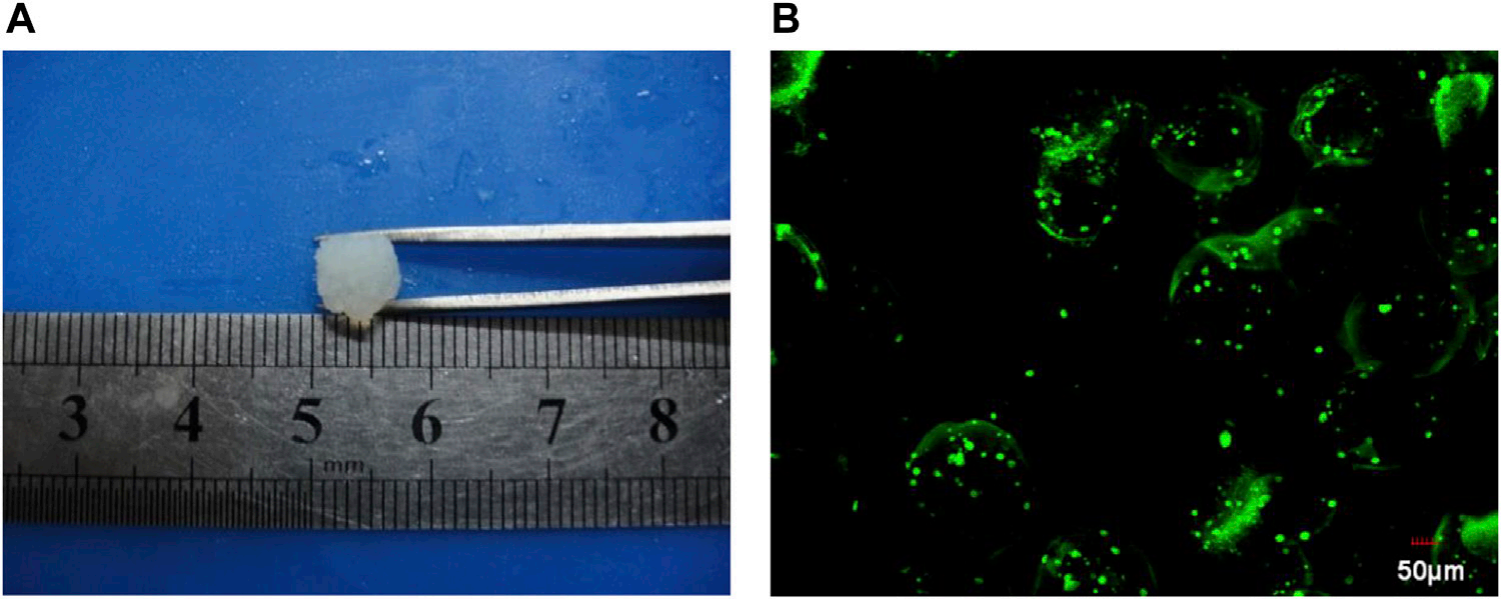
The image of gel **(A)** and cell microencapsulation complex under confocal laser scanning **(B)**.

The prepared silk fibroin solution was mixed with the prepared microencapsulated BRL-3A cells to obtain a silk fibroin-microencapsulated cell complex, and a small-sized block composite was obtained by means of a mould as shown in Figure 6. From the laser confocal microscopy, it can be seen that the BRL-3A cells in the silk fibroin-microencapsulated cell complex had a good survival rate and can be further investigated.

### Detection of BRL-3A cell metabolites in complex

**FIGURE 7:**
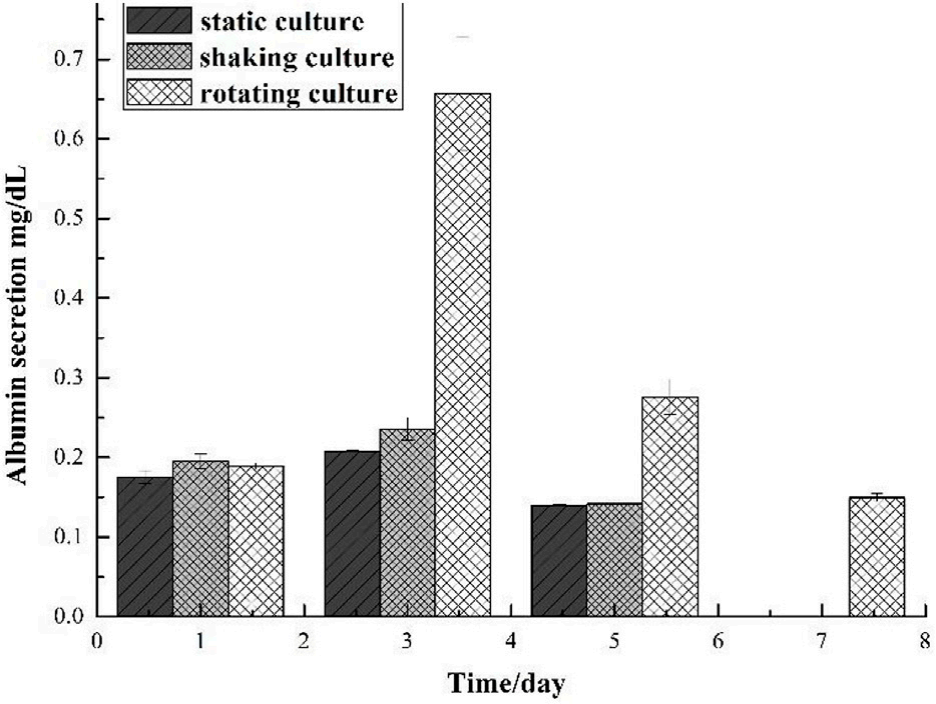
Comparison of albumin secretion of gel and cell microcapsulation composites in different culture system.

It can be seen from Figure 7 that on the first day, the content of synthetic albumin was not high, which is about 0.2 mg/dl; on the third day, the ability to synthesize albumin in the complex has been improved significantly under the microgravity culture system, while the complex in other culture systems has not increased the albumin content obviously; on the fifth day, the ability of the complex to secrete albumin in each culture system was greatly reduced, which indicated that the cell survival rate in the complex decreased; on the seventh day, only the complex under simulated microgravity culture can secrete a small amount of albumin, and the cells in the complex under other culture systems almost had no albumin secretion ability.

**FIGURE 8:**
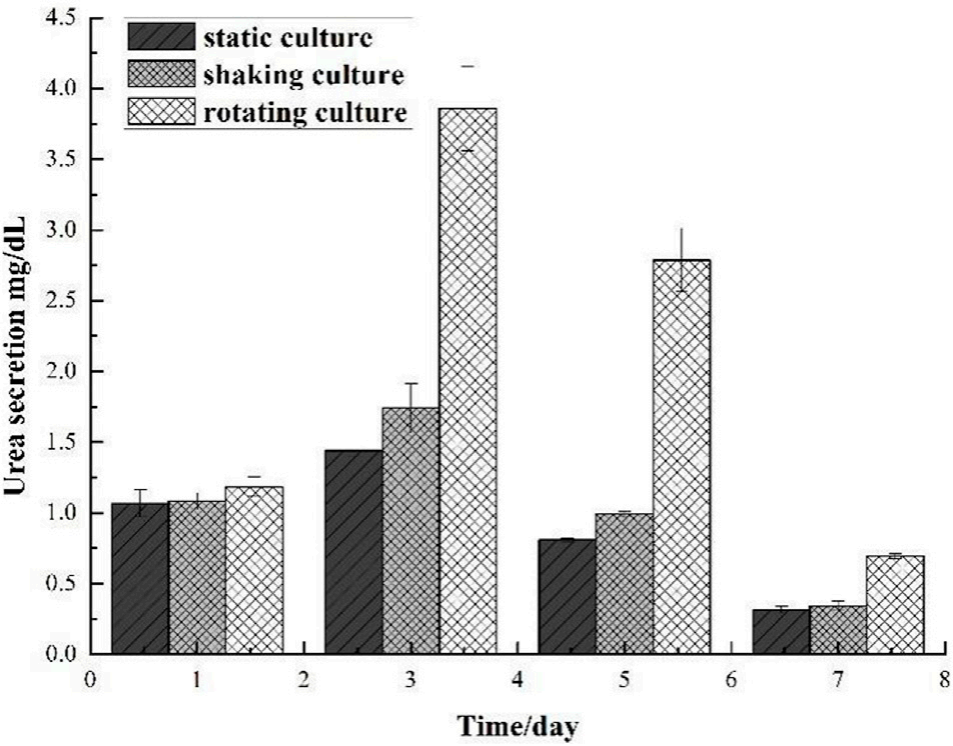
Comparison of urea secretion of gel and cell microcapsulation composites in different culture systems.

The amount of urea secreted in the silk fibroin gel-microencapsulated cell complex under different culture systems was shown in Figure 8, and the results were in accordance with the results of albumin. On the first day, the cells in the complex could secrete urea, and the content was about 1 mg/dl; on the third day, the ability to secrete urea in the complex under microgravity culture system was significantly improved, while the amount of urea secreted in the complex under other culture systems increased slightly; on the fifth day, the ability of the complex to secrete urea decreased in each culture system, indicating that the cell survival rate in the complex decreased; on the seventh day, only a small amount of urea was secreted, including the compound cultured under simulated microgravity.

Compare the albumin and urea secretion from the complex with those from the microcapsules, wen can see that the size plays an important role in mass transfer. For the silk fibroin gel-microencapsulated cell complex, the larger scale hinders the metabolites diffuse from the inside out.

## Conclusion

BRL-3A cells were cells with strong adaptability and rapid proliferation, which were easy to expand *in vitro* and had great expansion potential. The preparation of microencapsulated cells was carried out using the mature preparation process of the laboratory, and the results showed that microencapsulation did not affect cell survival. BRL-3A cells were cultured under simulated microgravity culture system, and the whole cell colonies showed a state of good cell growth. Under this culture system, the time of proliferative ability and metabolic capacity of the cells can be further extended on the basis of microencapsulated hepatocytes. Silk fibroin provided mechanical support for maintaining the structure of the gel-artificial cell complex. Since artificial cells are well distributed in the complex, the demands of high porosity and high connectivity of the traditional tissue engineering scaffolds can be avoided. This complex also has excellent mass transfer properties due to the inner liquification of the artificial cells. This work provides an alternative way for cell culture and tissue construction *in vitro*. However, much work should be done before this strategy being applied in the future. The main problem is that the secretions of albumin and urea from the complex are much fewer than those from the microcapsules. Thus, how to maintain the cell viability for a long period in the complex is a challenge. In the future work, we will attempt to build microvessels at the same time of the microtissue formation to enhance the mass transportation in the microtissues.

## Data Availability

The original contributions presented in the study are included in the article/Supplementary material, further inquiries can be directed to the corresponding authors.
